# Supplementary material for: Towards Personalised Contrast Injection: Artificial-Intelligence-Derived Body Composition and Liver Enhancement in Computed Tomography
Source: J Pers Med. 2021 Feb 24;11(3):159. doi: 10.3390/jpm11030159 (PMC7996171; doi:10.3390/jpm11030159)
Supplement: Supplementary file 1 [file jpm-11-00159-s001.pdf]

Journal of
Personalized Medicine

MDPI

Table 1. Correlations in regression.

Correlations	BMI (*r*)	BMI (*p*)	TBW (r)	TBW (*p*)	%LBW (*r*)	%LBW (*p*)
ΔHU/gI	-0.393	0.253	-0.531	0.000	0.323	0.493
ΔHU/(gI/TBW)	-0.645	0.139	-0.698	0.302	0.597	0.628
ΔHU/(gI/LBW)	0.190	0.099	0.152	0.371	-0.733	0.000

Correlations of ΔHU/gI, ΔHU/(gI/TBW) and ΔHU/(gI/LBW) with BMI, TBW and %LBW. Values are R-values and p-values. (TBW: total body weight; BMI: body mass index; LBW: lean body weight).

Table 2. Enhancement values per group.

Enhancement	Group ≤ 70 kg		Group 70 – 90 kg		Group ≥ 90 kg	
< 40 HU	0	0.0%	5	8.1%	1	5.0%
>70 HU	6	30%	3	4.8%	0	0.0%
< 50 HU	4	20%	20	30%	7	35%

Number of enhancement values < 40 HU, > 70 HU, and < 50 HU. Values are the number of patients and percentages are proportions of weight group.

Table 3. Subjective phase classification per rater.

	Timing (M.K.)		Timing (F.W.)	
Too early (non-diagnostic)	0	0.0%	0	0.0%
Early	0	0.0%	0	0.0%
Good	87	85.3%	90	88.2%
Late	15	14.7%	12	11.8%
Too late (non-diagnostic)	0	0.0%	0	0.0%

Subjective phase scores on a five-point scale of M.K. and F.W. in numbers and percentages of the total population.

Table 4. Subjective enhancement classification on the four-point Likert scale per rater.

	Enhancement (M.K.)		Enhancement (F.W.)	
Excellent	69	67.6%	68	66.7%
Good	29	25.5%	27	26.5%
Moderate	6	5.9%	7	6.9%
Non-diagnostic	1	1.0%	0	0.0%

Subjective enhancement scores on a four-point Likert scale of M.K. and F.W. in numbers and percentages of the total population.
